# Formaldehyde Reduction in an Operating Room Setting: Comparison of a Catalytic Surgical Vacuum Device With a Traditional Smoke Evacuator

**DOI:** 10.7759/cureus.38831

**Published:** 2023-05-10

**Authors:** Gregory T Carroll, David L Kirschman

**Affiliations:** 1 Scientific Affairs, Aerobiotix Inc, Miamisburg, USA

**Keywords:** catalysis, activated carbon, portable filtration, medical devices, air quality, operating room, vocs, surgical smoke, formaldehyde, electrosurgery

## Abstract

Introduction

Electrosurgery exposes healthcare workers to volatile organic compounds (VOCs) including formaldehyde. Adopting electrosurgical devices that catalytically transform formaldehyde to benign substances has the potential to improve safety in surgical settings.

Materials and methods

We compared the efficiency of formaldehyde removal of two medical devices. The first was a novel surgical vacuum (SV) device containing ultra-low particulate air (ULPA) filtration, activated carbon and catalytic transition metal oxide. The second was a commonly utilized handpiece evacuator (HE) that contained only mechanical filtration and activated carbon granules. Both devices were exposed to formalin vapor.

Results

The time weighted average (TWA), median and peak concentrations of detected formaldehyde at the outflow of the SV unit were 90% lower than the corresponding values detected at the outflow of the HE device (p = 0.0034). When catalytic material was added to the HE device, the detected formaldehyde concentration at the outflow was reduced by 55% (p = 2.9 x 10^-15^).

Conclusions

The catalytic SV device has the potential to considerably reduce formaldehyde levels in operating room (OR) environments.

## Introduction

Multiple toxic gaseous compounds are produced by electrosurgery which create occupational health concerns. These compounds include benzene, toluene, styrene, acrylonitrile, 1,3-butadiene, and formaldehyde [1,2]. However, a recent study specifically identifies formaldehyde as a unique concern with the greatest environmental increase during electrosurgical procedures where activated carbon filtration is utilized [3]. While activated carbon is employed as a general adsorbent to remove volatile organic compounds (VOCs) from the air [4], it has been acknowledged that activated carbon is limited in its ability to remove formaldehyde [5-8]. This is likely due to the polar molecular structure of formaldehyde, which does not interact strongly with the largely aromatic surface of activated carbon compared to more conjugated and less polar VOCs. Incorporation of polar groups into the activated carbon surface increases its affinity for formaldehyde, however, in humid environments, such as in a surgical plume, competition with water adsorption reduces the efficiency of formaldehyde capture [8].

Formaldehyde is a volatile, toxic compound that is associated with various health complications including cancer [9]. The permissible exposure limit set by the United States Occupational Safety Health Administration (OSHA) is 0.75 ppm as an 8-hour time-weighted average (TWA) and the short-term exposure limit is 2 ppm as a 15-minute TWA. The action level (trigger point for increased monitoring and medical surveillance) is 0.5 ppm as an 8-hour TWA. 20 ppm is considered to be immediately dangerous to life and health by the National Institute of Safety and Health [10].

In order to reduce exposure to surgical smoke, smoke-evacuating technologies have been developed [2,11]. Handpiece evacuators (HE) contain suction tubing attached to an electrosurgical stylus, which allows some of the smoke generated during electrosurgery to be re-directed to an ultra-low particulate air (ULPA) filter and activated carbon cartridge. These devices are designed for general purpose smoke removal, with an emphasis on removing visible smoke particulate. Their effectiveness at removing specific airborne pollutants such as formaldehyde is not well understood. Additionally, some surgeons have resisted the adoption of HE devices due to compromised ergonomics, distraction, and noise [12]. Recently reported novel surgical vacuum (SV) devices offer an alternative mode of smoke evacuation that is not limited by the dimensions of the surgical handpiece, does not compromise a surgeon’s dexterity, and has a higher relative air flow rate [5,13]. Additionally, the SV device reported uses a transition metal catalyst [14,15] that transforms formaldehyde to H_2_O and CO_2_.

There is currently a lack of understanding and awareness regarding the efficiency of electrosurgical smoke evacuation technologies in removing formaldehyde. In order to better understand the practical differences between a traditional HE that uses ULPA/activated carbon granules and a catalytic portable filtration SV device that uses ULPA/activated carbon combined with a formaldehyde oxidizing catalyst, we performed a controlled test that monitors the concentration of formaldehyde in the air outflowing from each device after uptake of formaldehyde vapor. Additionally, we modified a traditional HE device with a catalytic material and compared formaldehyde emission with and without the catalyst. 

## Materials and methods

Formaldehyde detection

Testing was performed in a 50 m^3^ simulated operating room (OR) previously described [16]. The room was equipped with typical OR airflow obstructions including surgical lights, tables, and medical equipment. Formaldehyde was introduced into the air stream via evaporation from a formalin (formaldehyde in water) solution at room temperature. A calibrated gas-sensitive semi-conducting formaldehyde sensor (Aeroqual, Aukland, New Zealand) [17] was used to track formaldehyde levels in the OR. The sensor measures from 0-10 ppm with an accuracy of < ±0.05 ppm within the range of 0-0.5 ppm and ±10% within a range of 0.5-10 ppm. The accuracy is not specified above 10 ppm. The sensor has a resolution of 0.01 ppm. Readings were taken every minute. For each experiment, the sensor was placed at the air outlet of the device tested.

Air decontamination devices

**Figure 1:**
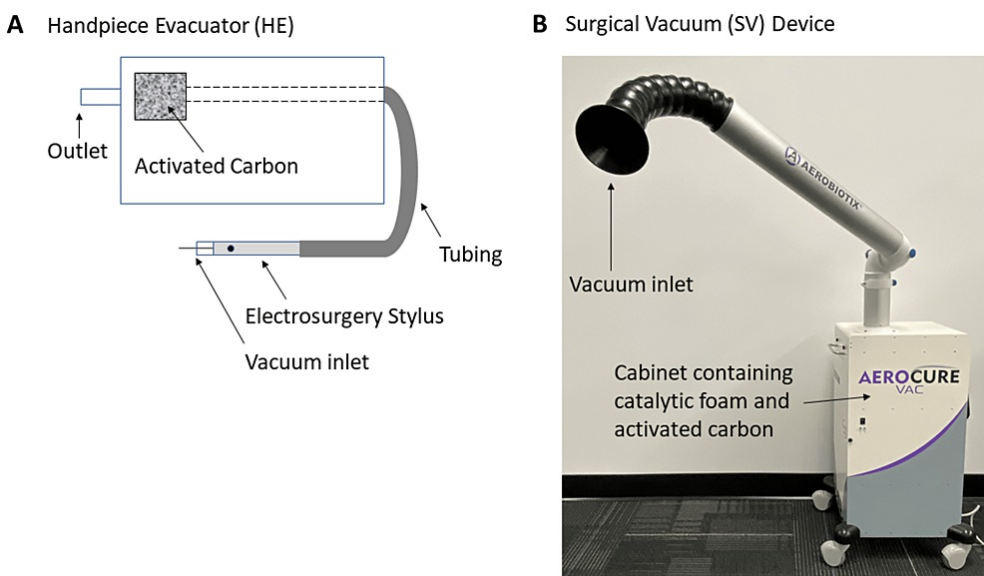
The two devices tested were a traditional handpiece evacuator (HE) with a base unit and electrocautery handpiece connected by suction tubing (A) and a self-contained surgical vacuum (SV) unit (B). HE: Handpiece Evacuator SV: Surgical Vacuum

Two devices were compared (Figure 1A-1B). The first was a representative traditional smoke HE. The device contained activated carbon granules for VOC removal. The vacuum inlet of the device was built into the electrosurgery pencil in order to remove surgical smoke at the site of generation. The diameter of the vacuum inlet was approximately 1 cm. The second device tested was a portable filtration SV unit (Aerobiotix, Aerocure Vac, Miamisburg, USA). The device has a noise level of 62 dB at 1 m from the outlet and a flow rate of 2.0 l/s. The device was equipped with a 25 x 25 x 1.3 cm activated carbon filter for adsorbing VOCs. A proprietary foam embedded with a transition metal oxide catalyst for converting formaldehyde to CO_2_ and H_2_O was placed behind the activated carbon filter so that incoming air first passes through the activated carbon. The device also contains a particulate matter filter and ultraviolet lamps for air disinfection.

Formalin experiments

The air inlet of each device was placed over a bottle of open formalin (approximately 20 ml) for 5 minutes. After 5 minutes each device continued to run for an additional 5 minutes without formalin exposure. Formaldehyde levels were measured before, during, and after formalin exposure. An additional test was performed in which a piece of the catalytic foam technology in the SV device was placed in the HE device. The material was cut to the dimensions of the HE cavity containing the activated carbon granules and placed behind the carbon granules cartridge so that formaldehyde vapors passed through it prior to outflow from the device. 1 ml of formalin was placed in a capped plastic cup containing an outlet. The cup was attached to the HE device via Tygon tubing (Saint-Gobain S.A., Courbevoie, France) and vacuum was pulled. Formaldehyde concentrations at the outlet of the device were recorded for 1 hour of exposure.

## Results

**Figure 2:**
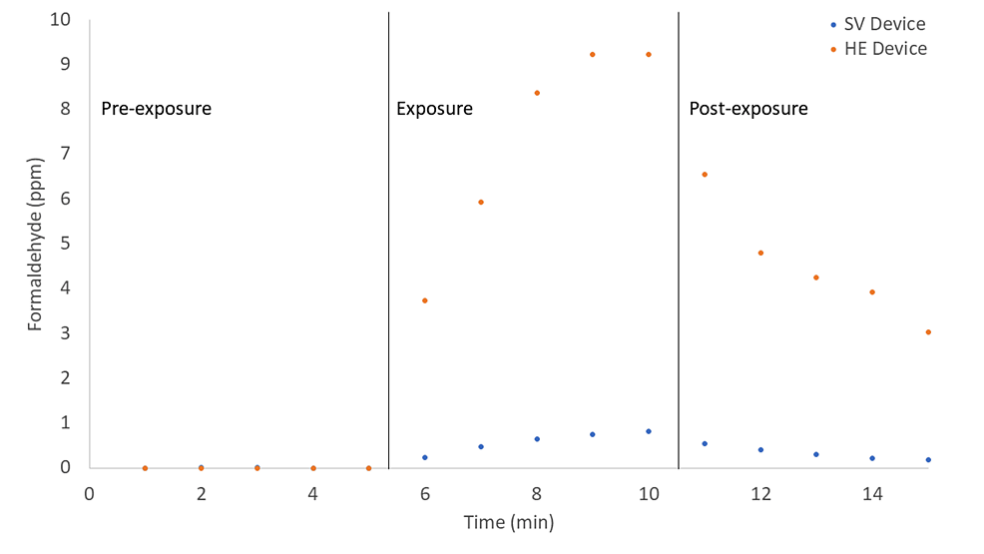
Formaldehyde concentrations as functions of time at the air outlets for a catalytic SV device (blue) and a traditional HE unit (orange) exposed to formalin for 5 minutes. SV: Surgical Vacuum HE: Handpiece Evacuator

Both an SV device and a traditional HE unit were exposed to an open bottle of formalin for five minutes. Detected formaldehyde values before, during, and after exposure are presented in Figure 2. During exposure, the concentration of formaldehyde at the outlet increased for both devices. The concentration increased much more rapidly and reached significantly higher values for the traditional smoke HE which contains carbon granules as an adsorbent for VOCs. The highest value detected for the traditional evacuator was 9.23 ppm, which occurred after four and five minutes of exposure. The concentration of formaldehyde emitted decreased to 3.03 ppm five minutes after exposure was stopped with the vacuum continually running. The highest value detected for the SV device was 0.82 ppm, which occurred after 5 minutes of exposure. The concentration of formaldehyde emitted decreased to 0.18 ppm five minutes after exposure was stopped with the vacuum continually running. 

The TWA, standard deviation (SD), median, and peak formaldehyde concentrations detected during and after formalin exposure are presented in Table 1. The TWA (SD), median and peak concentrations for the HE unit are, respectively, 5.90 (2.35), 5.36, and 9.23 ppm. The corresponding values for the SV device are, respectively, 0.46 (0.23), 0.44, and 0.82 ppm. The TWA is reduced by 92% when the SV device is used in place of the HE device.

**Table 1:** TWA, TWA percent reduction, SD, median, and peak formaldehyde concentrations (ppm) detected at the outlets of the HE and SV devices. HE: Handpiece Evacuator SV: Surgical Vacuum TWA: Time-weighted Average SD: Standard Deviation

Device	TWA (ppm)	TWA % Reduction	SD (ppm)	Median (ppm)	Peak (ppm)
HE Device	5.90	---	2.35	5.36	9.23
SV Device	0.46	92	0.23	0.44	0.82

**Figure 3:**
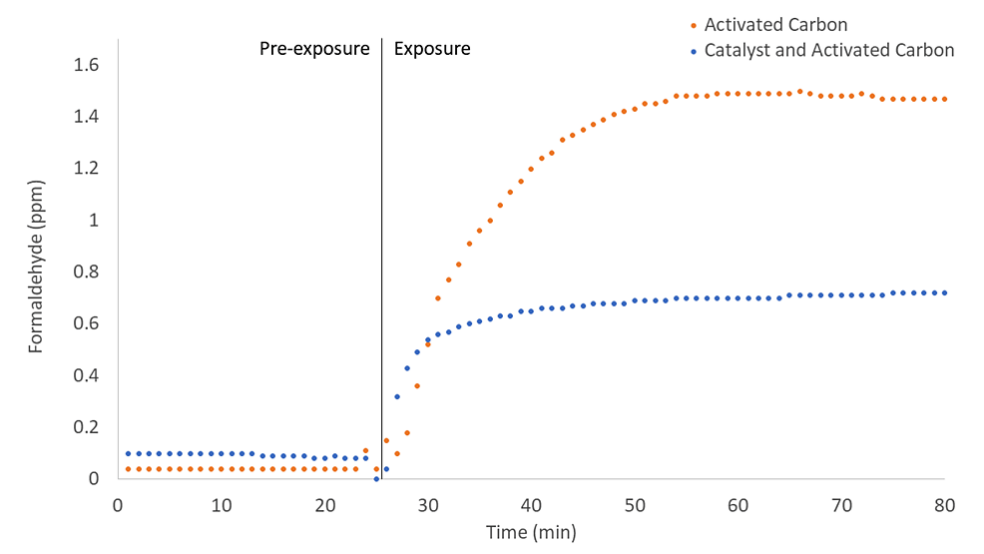
Formaldehyde concentrations as functions of time at the air outlet for an HE device containing: activated carbon (orange); catalyst and activated carbon (blue). HE: Handpiece Evacuator

The catalyst technology in the SV device was placed in the HE device in order to test its efficacy compared to carbon granules under identical conditions. A capped container containing 1 ml of formalin was interfaced with the HE device so that the vacuum pulled directly from the container. The volume of formalin was kept small to prevent the outflow saturation of the detector. Vacuum was applied to the container for 1 hour. The concentrations of emitted formaldehyde at the air outlet of the device with and without catalyst as functions of time are shown in Figure 3. When only carbon granules were present, the formaldehyde concentration reached a stable maximum value of 1.64 ppm. When the catalyst was added to the device, the maximum value reached only 0.64 ppm, which is a 55% reduction in the concentration of emitted formaldehyde compared to when only carbon granules are present.

## Discussion

Our results show that the catalytic SV device is more efficient at removing formaldehyde than the traditional HE unit. The SV device outflow emits lower concentrations of formaldehyde compared to the HE device. The TWA, median, and peak formaldehyde concentrations of the SV device are all less than 1/10th the concentrations emitted by the HE. The SV device contains both activated carbon and a catalytic material that transforms formaldehyde into CO_2_ and H_2_O [18]. The HE contains only activated carbon granules and can only remove formaldehyde via adsorption. The strength of the binding between the activated carbon granules and the formaldehyde is uncertain. Previous reports indicate that in air cleaning devices activated carbon shows little, if any, capacity to remove formaldehyde from the air [5, 6]. It is well-known that adsorbates can desorb, which limits the efficiency of adsorption-based VOC removal [7]. While there might be some binding interactions between the surface of the carbon granules and the formaldehyde in the air stream, the activated carbon is not expected to contain the formaldehyde indefinitely. The presence of a constant airflow facilitates desorption. After the removal of the formaldehyde source, the formaldehyde levels decrease, however, over a five-minute interval the concentration of emitted formaldehyde for the HE device does not reduce to the level of the peak concentration observed for the SV device. The relatively high levels of formaldehyde for the HE device even after removal of the source suggests that formaldehyde is desorbing from the activated carbon granules as an unpolluted air stream constantly flows through the device. However, the efficiency of the SV device in a clinical setting needs further investigation.

When the catalytic material is placed in the HE, the amount of emitted formaldehyde decreases by 55%. The reduction in emitted formaldehyde when only the catalytic material is added is further evidence for the improved performance of catalytic removal in comparison with adsorptive removal. Our results show that the use of the SV device, which contains both activated carbon and a catalytic material that transforms formaldehyde into benign substances, is a more efficient method to remove formaldehyde from indoor air compared to the HE device which only employs activated carbon. 

There are some limitations of our study. Formaldehyde was produced via evaporation from formalin, rather than an electrosurgical procedure, which contains additional components including water vapor, particulate matter, and many different VOCs. The quantity and rate of formaldehyde entering the devices are expected to differ from actual surgical procedures. The actual distances between the devices and the surgical site will not be exactly the same. The traditional device is closer to the site given that it is interfaced with the electrosurgical stylus, however, in practice the surgical vacuum is covered with a disposable sterile drape, allowing surgeons to bring the adjustable vacuum nozzle as close as possible to the surgical site. The surgical vacuum nozzle has an inflow diameter of approximately 11.4 cm and an airflow of 100 CFM (Cubic Feet Per Minute). Handpiece devices typically have inflow diameters of approximately 1 cm and an airflow of 20 CFM. While the vacuum inlet of the HE is ultimately closer to the surgical site, the vacuum inlet of the SV has a stronger airflow and larger inflow diameter. Confounding factors including temperature and humidity could potentially affect the performance metrics of the devices. Clinical studies will need to be performed to compare the impact of the devices during electrosurgery on human tissue during an actual surgical procedure.

## Conclusions

We have shown that an SV device containing a catalytic material shows lower concentrations of formaldehyde at the outflow of the unit compared to a traditional HE device. The levels of detected formaldehyde when the SV device was tested were more than 90% lower compared to the HE device. When the catalytic material was placed in the HE device, the levels of emitted formaldehyde were reduced by 55%. The combination of catalytic material and activated carbon is superior to activated carbon alone for formaldehyde removal. Utilization of the SV device during surgical procedures is expected to improve air quality in ORs. 
